# Non‐glandular findings on breast ultrasound. Part II: a pictorial review of chest wall lesions

**DOI:** 10.1007/s40477-022-00773-1

**Published:** 2023-01-27

**Authors:** Antonio Corvino, Orlando Catalano, Carlo Varelli, Giulio Cocco, Andrea Delli Pizzi, Fabio Corvino, Corrado Caiazzo, Domenico Tafuri, Martina Caruso

**Affiliations:** 1 Movement Sciences and Wellbeing Department, University of Naples “Parthenope”, Via Medina 40, 80133 Naples, Italy; 2 Radiology Unit, Varelli Diagnostic Institute, Naples, Italy; 3 Unit of Ultrasound in Internal Medicine, Department of Medicine and Science of Aging, University “G. D’Annunzio”, Chieti, Italy; 4 Departiment of Innovative Technologies in Medicine and Dentistry, University “G. D’Annunzio”, Chieti, Italy; 5 Vascular and Interventional Radiology Department, Cardarelli Hospital, Naples, Italy; 6 Radiology Unit, PSP Corso Vittorio Emanuele ASL Napoli 1, Naples, Italy; 7 Advanced Biomedical Sciences Department, University Federico II of Naples, Naples, Italy; 8 via B. Croce n. 82, 81033 Casal di Principe, CE Italy

**Keywords:** Breast ultrasound, Chest wall lesions, Characterization, Differential diagnosis, Pitfalls

## Abstract

The breast ultrasound (US) field-of-view (FOV) includes glandular parenchyma as well as tissues located anterior to and posterior to it, up to pleural line. For that, it is possible to incidentally identify lesions unrelated to breast parenchyma during screening or diagnostic US; sometimes a palpable lump may be the reason of the imaging exam. Furthermore, abnormality related to chest wall are easier and more accurate detected after mastectomy. Hence, radiologists should know the US appearance of lesions which may develop from all tissues present in this region and displayed in the US FOV, without focusing only on glandular abnormalities while performing the exam. This is the second of a two-part series on non-glandular breast lesions; in detail, part two provide an overview of US appearance, differential diagnosis, and pitfalls of chest wall lesions. They may have an infectious, traumatic, inflammatory etiology or be benign or malignant neoplasms. The US role in the assessment of chest wall abnormalities is limited, usually computed tomography and/or magnetic resonance are requested as second-level imaging exams to characterize and to assess better their relationship with surrounding structures because of larger and panoramic view. Finally, US could be useful to guide biopsy.

## Introduction

This is a second part of a two-part series on abnormal findings unrelated to the glandular parenchyma, which can be found when performing an ultrasound (US) examination of the breast, as the tissues located anterior and posterior to it are visualized in the US Field of View (FOV). The resulting lesions from these tissues can be clinically detected, misdiagnosed as a breast lump, and thus be the reason for the examination or they can be an incidental finding at US. The role of the radiologist is to identify the lesion, to evaluate the relationship between it and the surrounding tissues in order to recognize whether it derives from glandular parenchyma, superficial layers or chest wall. First of all, it is important to know the anatomical composition of the breast region and the US appearance of any anomaly. In the first part we illustrated the broad spectrum of superficial non-glandular entities that can develop in tissues located anterior to the mammary glandular parenchyma, while chest wall lesions are examined in this manuscript.

The chest wall is made up of fat, nerves, blood and lymphatic vessels, muscles, bones, cartilage, and fibrous connective tissue, so injuries may result from any of these component tissues. Various pathological conditions can occur, such as infections, inflammation, benign or malignant neoplasms. Knowledge of their clinical and US characteristics as well as an in-depth anatomical knowledge are necessary for the correct evaluation: the diagnostic and/or therapeutic management varies according to the nature and location. In particular, deeper lesions originating from the chest wall should require computed tomography (CT) and/or magnetic resonance (MR) as second level imaging exams to better characterize and evaluate their relationship with surrounding structures due to the wider and more panoramic view. Additionally, US could be helpful in guiding biopsy. In the case of a mastectomy, the US assessment of chest wall is simpler and more accurate because of the absence of mammary glandular parenchyma.

The purpose of our manuscript is to illustrate the broad spectrum of non-glandular entities that can develop in the chest wall and their US findings, focusing on the integrated diagnostic role of color-Doppler imaging technique, highlighting pitfalls and differential diagnoses.

## Sonographic anatomy of the chest wall

In the deep portion of breast US FOV, chest wall is partially visualized: the knowledge of its US appearance and its pathological conditions should be carefully known by radiologists. First of all, optimizing the ultrasound technique is essential: adjustments to deep portion in the FOV, focal zones, time gain compensation, dynamic range, and post-processing gray scale imaging can improve the imaging quality, and the lesions can be more clarified. Transducers of a lower frequency, such as 5 MHz, may be helpful, even though the lower resolution may reduce the accuracy of lesion evaluation.

Furthermore, the chest wall anatomy should be well known. At the level of mammary region, chest wall is visualized deeper than retromammary zone [1]. In detail, in the upper part of this region, the most superficial chest wall structure is the pectoralis major muscle, with intercostal and serratus anterior muscles, ribs, and costal cartilages lying more deeply. In the inferior segments of the breast, the pectoralis muscle is not present, and upper abdominal musculature and serratus anterior muscles may be the first encountered [2]. The muscular echotexture is characterized by multiple echogenic striae on longitudinal scans or multiple echogenic dots on transverse scans over a hypoechoic background. On US only the anterior cortex of the ribs is evaluable, it appears as a continuous smooth bright interface with marked posterior acoustic shadowing, whereas costal cartilage is visible as a homogenous and less echogenic structure than the adjacent muscle, round or ovoid on a longitudinal image, tubular on a transverse one. Finally, the two layers of the pleura are seen as one thin line over the bright interface with the lung [2].

## US imaging findings of chest wall lesions

**Fig. 1:**
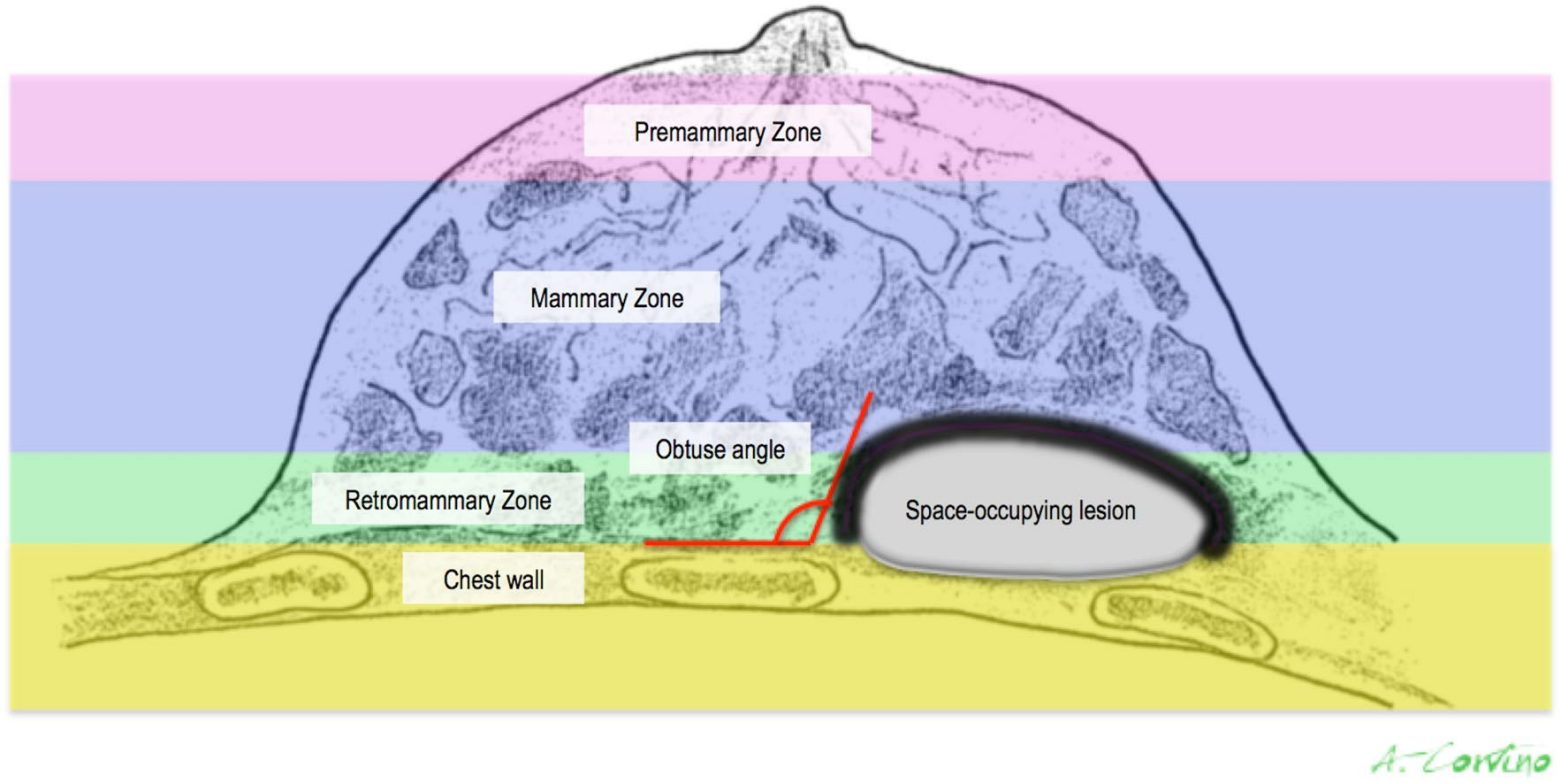
Schematic picture of the breast region: pre-mammary zone (pink), mammary zone (violet), retro-mammary zones (green) and chest wall (yellow). A space-occupying lesion originating from chest wall structures displaces retro-mammary fat anteriorly (black line) and forms an obtuse angle to the chest wall

Few imaging findings may help in the identification of the origin layer of an abnormal finding displayed in the deeper portion of the US FOV [3, 4]. First of all, lesions that develop deeply in the pectoral fascia should be considered non-glandular until proven otherwise. In particular, those originating from deep structures displace breast tissue anteriorly, as they expand into the breast, and an obtuse angle to the chest wall may be identified. The presence of a retro-mammary fat layer anteriorly to the lesion is in favor of extra-mammary non-glandular origin (Fig. 1). In daily clinical practice, lumps of the chest wall are frequent: painful or not, of long standing or of recent appearance, constant or intermittent. However, the clinical relief does not always correspond to a pathologic abnormality, since it may be due to conformational asymmetries, such as related to muscles, bony protrusions, inversion of the xyphoid appendix or costal dysmorphism. On the other hand, some pathological lesions are not palpable, especially if the subcutaneous fat layer is thick. In the differential diagnosis of anterolateral chest wall lesions, either those specific of this region or those also detectable elsewhere should be considered: bursitis of scapulo-thoracic and subscapular bursa, fluid collections, inflammatory conditions, benign or malignant lesions arising from superficial soft tissue, bone or cartilage. In addition to these expansive/infiltrative injuries, osteoporotic or traumatic rib fractures, which can cause mastodynia, and focal or diffuse changes in the pectoral muscle must also be considered. In the assessment of chest wall lesions, CT and/or MR should be requested as second-level imaging technique because of their panoramic view; in addition, the intravenous administration of contrast medium allows an accurate characterization and assessment of relationship with surrounding tissues.

### Neurogenic tumors: schwannoma and neurofibroma

**Fig. 2:**
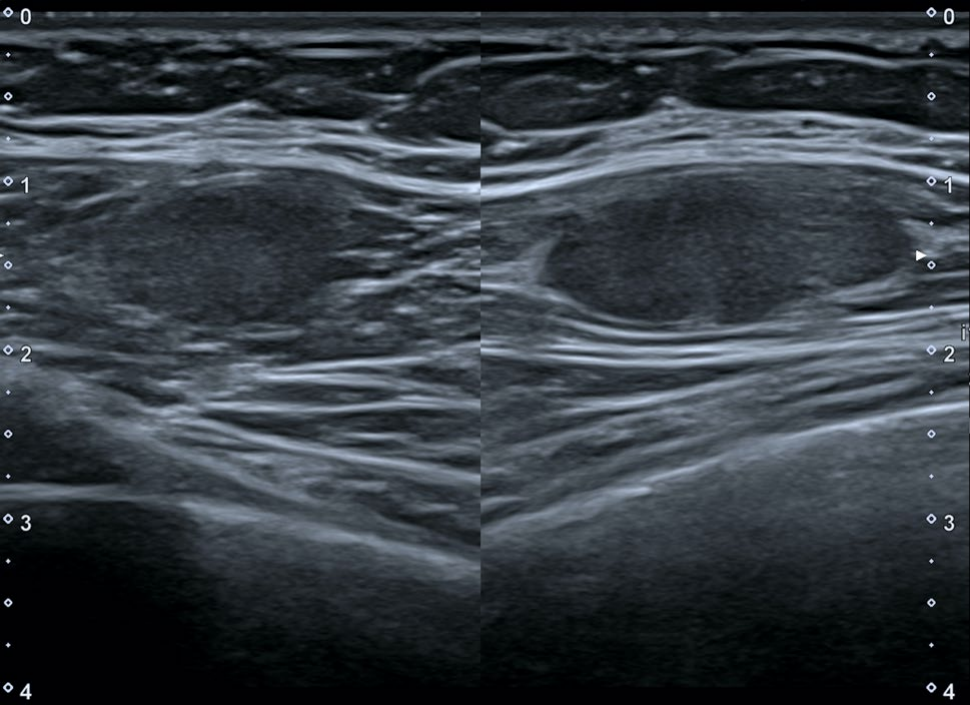
Female patient of 55 years old. B-mode scans. The US exam shows a well-defined, hypoechoic schwannoma of the chest wall with oval shape on longitudinal scan

**Fig. 3:**
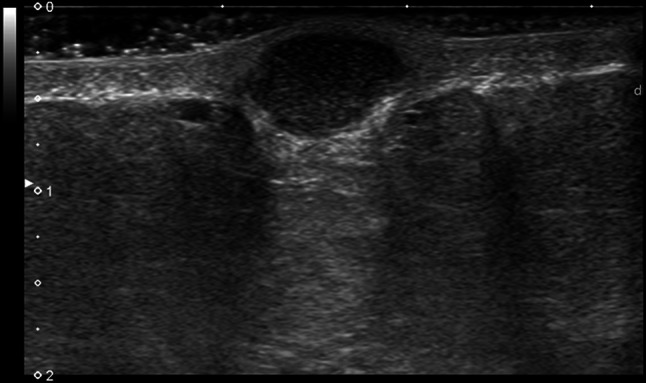
Female patient of 40 years old. B-mode scan. Neurofibroma of the chest wall in a patient with neurofibromatosis appears as a round, well-defined hypoechoic nodule, which displaces retro-mammary fat anteriorly

The two most common types of peripheral nerve sheath tumors are schwannoma, also known as neurilemmoma, and neurofibroma, which may present as a solitary or multiple mass as part of neurofibromatosis. In the chest wall they originated from the intercostal nerves and have been reported rarely and equally among women and men [5]. First of all, a presumptive diagnosis of neurogenic tumor can be reliably made with US if a soft tissue mass is found to be connected to a nerve bundle at its proximal and distal poles. The visualization of small nerve branches may be difficult, the nerve ends might be extrinsic to the lesion or distorted and stretched over the tumor capsule, thickened and present loss of fascicular structure, so that a careful scanning technique is needed [6]. The imaging differential diagnosis between schwannoma and neurofibroma is difficult, because there are not high specific US findings, with the exception of eccentric location regarding to nerve-tumor position, which is typical of schwannoma (Figs. 2 and 3). The US appearance is variable: usually they present as homogeneous, hypoechoic and well-defined mass with a round or oval shape and peripheral nerve continuity. Intra-tumoral cystic changes, posterior acoustic enhancement as well as increased flow on color Doppler are suggestive of schwannoma [3]. Of note, an eccentric position allows to exclude the possibility of neurofibroma, while schwannoma can show either eccentric or central position. Schwannomas arise peripherally from the nerve sheath and have a capsule called epineurium, hence the nerve-tumor transition is clearly defined, while neurofibromas grow interstitially in the center of the nerve bundle within the endoneurium, they are not encapsulated, which causes the nerve-tumor transition to be infiltrative [7]. Neurofibroma commonly have a coarse echogenic hypo-vascular pattern [3] and they may show a target appearance characterized by a hyperechoic center and a hypoechoic outer zone [8] as result of a dense fibrocollagenous center and a high fluid content myxoid periphery. The size may aid to differentiate these two entities: Ryu et. al. proposed a cut-off value greater than 4.00 mm as suggestive of neurofibromas [9]. Although rare, malignant transformation can occur with both of them, usually sarcomatous transformation of neurofibroma [6]: a sudden increase in size, indistinct tumor margins and adhesion to surrounding tissues are the suspected US findings.

### Rib fracture

**Fig. 4:**
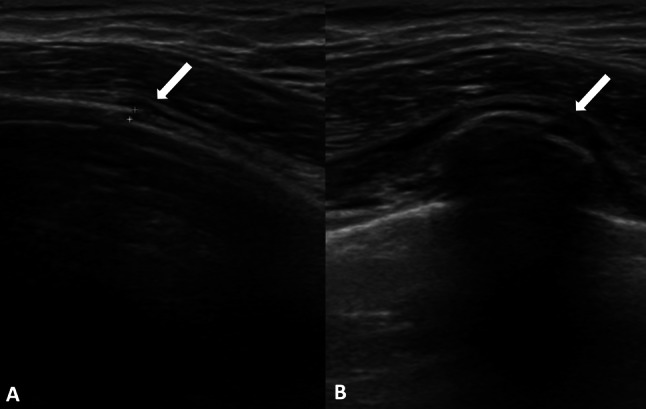
Female patient of 66 years old. B-mode longitudinal (**A**) and transverse (**B**) scans. Rib fracture (white arrow) mild displaced in a patient with localized mastodynia

**Fig. 5:**
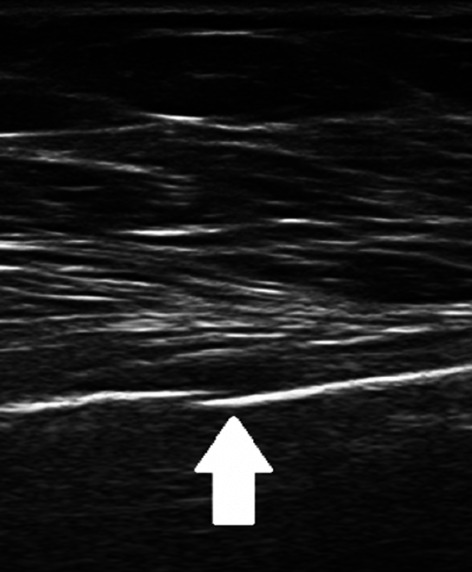
Female patient of 79 years old. B-mode longitudinal scan. Mild displaced rib fracture appears as a clear disruption of the anterior echogenic costal margin (white arrow)

Rib fractures are the most common (25%) injuries resulting from blunt chest trauma and they are usually revealed on radiographs, but some are missed, especially those occurring in costal cartilage [10]. Clinically they are suspected based on patient’s history and pain accentuated with inspiration and cough. The misdiagnosed rib fracture may cause a referred mastodynia, which can be the reason of breast US. As mentioned-above, the anterior margin of the costal cartilage and osseous rib is normally seen as a thin and continuous echogenic line, although a narrow discontinuity without a step may be seen at costocondral junction in healthy patients. The costal cartilage appears relatively hypoechoic compared with the osseous rib. Fractures were denoted by a clear disruption of the anterior echogenic margin: a non-displaced fracture was defined as a break without displacement, whereas displaced fracture as a break with displacement, which may be mild (< 1 mm), moderate (1–4 mm) or severe (> 4 mm) (Figs. 4 and 5) [11]. Indirect US findings of rib fractures are reverberation artifacts also known as “light house phenomenon” or “chimney phenomenon”, local hematoma, soft tissue swelling, pleural effusion and pneumothorax [10]. During the acute healing phase, increased echogenicity representing callus formation is seen and, over time, callus calcification appears as a small acoustic shadow with slight contour abnormality [12].

### Fatty degeneration of the pectoral muscles

**Fig. 6:**
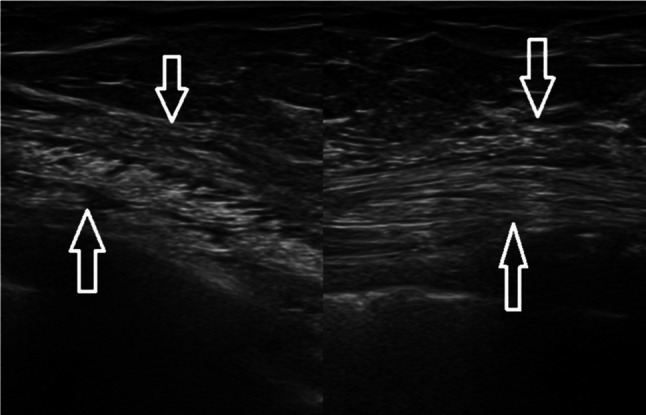
Female patient of 61 years old. B-mode scans. Adipose degeneration of the pectoral muscles is characterized by an increased echogenicity and decrease of the thickness (white arrows)

The muscle fatty degeneration is usually due to muscle atrophy, it is associated with lack of muscular mobility and with major co-morbidity such as obesity, osteoporosis and type 2 diabetes. In the literature, it was widely described for the rotator cuff lesions and supraspinatus muscle [13, 14], but fewer are articles about pectoral muscles [15]. In particular, Kotti et al. suggested the fatty degeneration of pectoralis muscles as a complication related to breast implants: the implant weight and the mechanical pressure on the fibers led to a cellular stress and a distortion in the regenerative process [15]. The real incidence of this disease is not known, because data presented in the literature is poor and limited to clinical observations. The US changes of muscular fatty degeneration are increased echogenicity from slight to marked compared with other muscles and also a decrease of the thickness may be observed (Fig. 6) [16].

### Malignant chest wall neoplasms

Neoplasms of the chest wall are uncommon and represent approximately 5% of all thoracic malignancies [17]. More than 50% of them are malignant and typically result from direct invasion by or metastasis from thoracic tumor, whilst the remaining are primary chest wall tumors, benign or malignant, arising from osseous structure or soft tissues [17]. Even though sarcomas are the most common primary malignancies of the chest wall (45% of which from soft tissue and 55% from bone), they are considered rare [18]. Patients may be asymptomatic or symptomatic, usually they suffer of chest pain. As stated before, US has a limited role in the evaluation and characterization of superficial chest wall lesions, but since this region is partially included in the breast US FOV, radiologists should know the US features of chest wall neoplasms in order to recognize them either as incidental findings or as non-glandular lesions, which could explain mastodynia. CT and/or MR should be suggested as second-level imaging exam. In detail, CT reveals a lesion’s presence, site and tissue origin (bone, cartilage or soft tissue), morphologic features, and internal components, such as fat and mineralization [19]. The intravenous administration of contrast material is useful to evaluate the tumor vascularity. On the other hand, since MR has superior soft-tissue contrast compared to CT, it represents the optimal imaging modality for delineating extent of chest wall soft-tissue involvement [20]. Furthermore, US can be used to guide needle biopsy.

Among primary soft tissue chest wall neoplasms, undifferentiated pleomorphic sarcoma is the most common [18]. It appears as a hypoechoic mass with a base along the chest wall, involves the deep fascia or skeletal muscles. Small lesions tend to be homogeneous, whereas large masses usually have a heterogeneous internal structure for the presence of necrotic or cystic areas, irregular and infiltrative margins, calcifications may also be seen [3]. These tumors have a poor prognosis: grade and differentiation are the most important factors affecting survival. Wide resection is the treatment of choice, and adjuvant therapy is considered for high-grade sarcomas. For these reasons and since local recurrences occur in 7–52% of cases, an accurate assessment of the tumor extension is requested before surgical excision. Regarding the diffusion in depth, CT and MR are the preferred imaging techniques, whereas US is considered superior to evaluate the superficial diffusion because of its better resolution of superficial layers. Furthermore, US is more accurate in recognizing the small satellite nodules, which are important to identify pre-operatively in order to accurately plan the surgical resection [18]. On CT and MR, sarcomas appear as area of soft tissue density/signal intensity often associated with internal necrotic areas of low density or high signal intensity on T2 sequences [20].

**Fig. 7:**
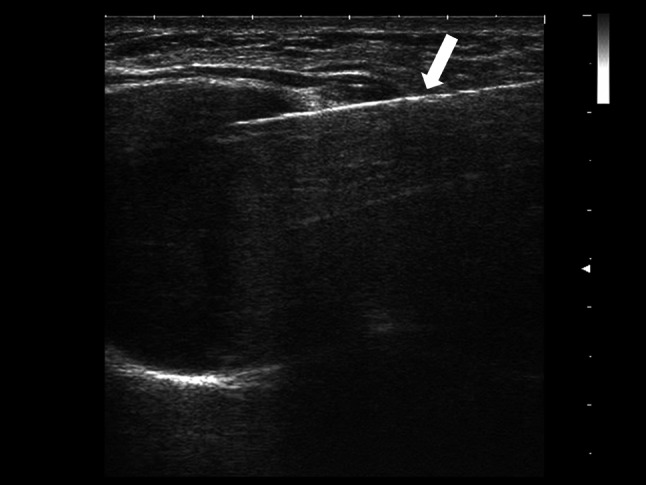
Female patient of 38 years old. B-mode scan during ultrasound guided core-needle biopsy (arrow). Chest wall sarcoma appears as a homogeneous, hypoechoic mass with irregular and infiltrative margins

Chondrosarcoma is the most common primary osseous malignancy of the chest wall, representing 33% of all primary rib neoplasms [21]. It also may be associated with malignant degeneration of benign chondromas, trauma, and thoracic radiotherapy. Approximately 10% of chondrosarcoma occur in the chest wall, mainly in the anterior chest wall, in the superior five ribs, adjacent to costochondral junctions, for that patients present a palpable and painful anterior chest wall mass, which can be misdiagnosed as breast nodule at clinical examination [22]. On US, a hypoechoic mass with irregular margins replaces the normal echogenicity of the rib, internal calcifications are usually seen (Fig. 7) [3].

**Fig. 8:**
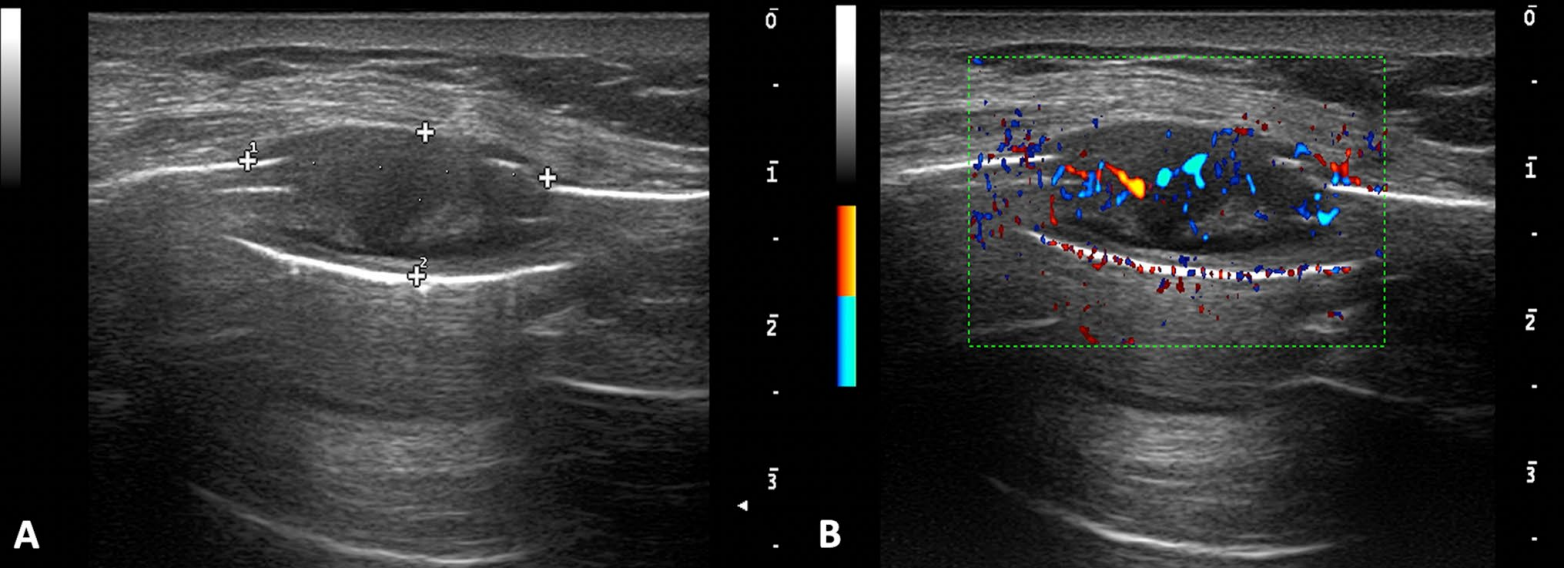
Female patient of 55 years old. B-mode (**A**) and color Doppler (**B**) scans. US reveals a heterogeneous, hypoechoic rib metastasis in patient with mastectomy, which disrupts echogenic cortical line. Color Doppler shows rich intralesional vascularization (**B**)

**Fig. 9:**
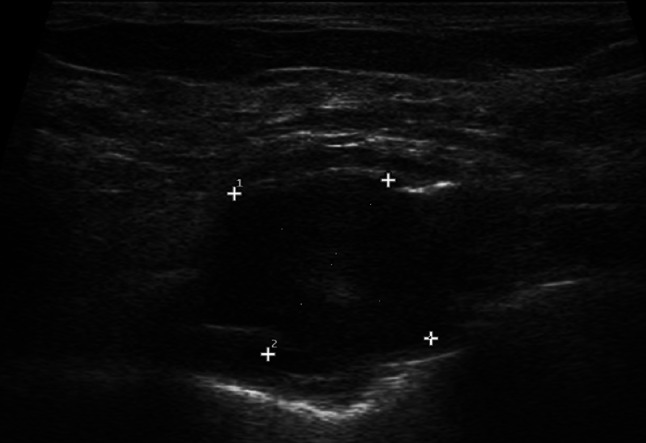
Female patient of 41 years old. B-mode scan. A myeloma rib lesion which clinically presents as a breast lump, whereas US shows a hypoechoic mass with irregular margins, replacing the normal echogenicity of the rib

**Fig. 10:**
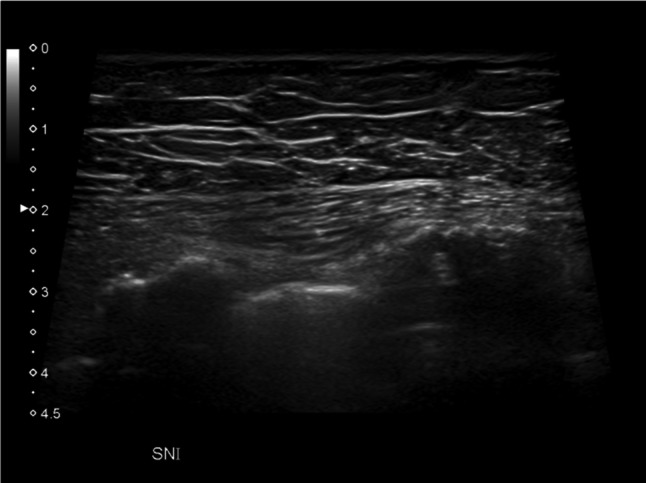
Female patient of 65 years old. B-mode scan. Irregular echogenic cortical line of ribs due to metastasis of breast cancer

**Fig. 11:**
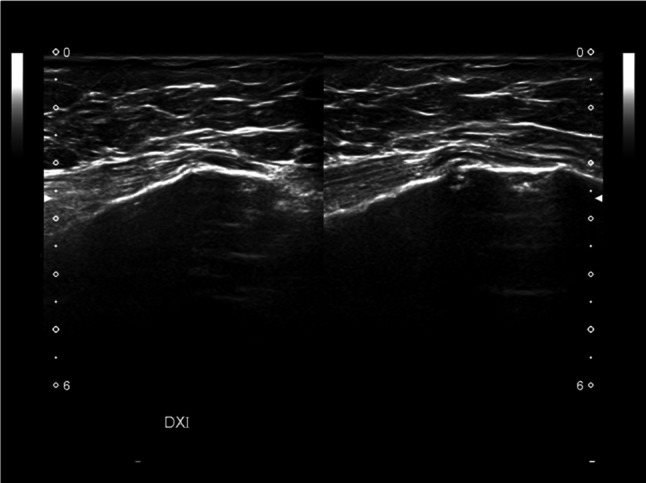
Female patient of 59 years old. B-mode scans. Irregular echogenic cortical line with focal disruption due to pathological fracture in a breast cancer metastatic patient

The most common chest wall malignancies are metastatic tumors, resulting from hematogenous or lymphatic dissemination of breast, lung, kidney, and prostate carcinomas [3]. Even though, bone metastases are better detected on bone scintigraphy and FDG-PET, they are visualized on US more easily than expected. The location of the pain aid in their US identification, they usually appear as lytic lesion of one or more ribs [23]. Longitudinal and transverse scans of the entire involved rib is mandatory. The cortical bone structure is destroyed, so the uniform echogenic thickness of the cortex is replaced by an irregular, thickened or disrupted echogenic cortical line, which may be associated with abnormal acoustic transmission. Infiltration of the bone appears as a hypoechoic mass, replacing the normal echogenicity of the rib (Figs. 8, 9, 10) [12]. Furthermore, because cancer patients are often elderly and/or cachectic, they may relatively easily suffer rib fractures after minor trauma, or even after coughing or straining. In these cases, US may show a clear disruption of the cortical echogenic thickness as a “step” or angulation; hematoma of the adjacent soft tissues may be associated (Fig. 11).

Metastatic lesions to the chest wall soft tissue are uncommon, usually seen in patients with extensive metastases elsewhere. Melanoma cancer is the most common cause [24]. They are evident as nodules with smooth or lobulated contours, hypoechoic compared with muscle, and sometimes with heterogenous echotexture for the presence of small anechoic areas [25]. The color-Doppler assessment reveals internal flow in the 70% of cases [26]. CT or MR, panoramic techniques, are always required to stage the disease before treatment.

### Pleuro-pulmonary findings

In the deeper FOV of breast US the visceral and parietal portions of the pleura can be seen as echogenic lines deep to the ribs, so it is possible to detect their abnormal findings, such as pleural thickening, pleural or peripheral lung nodule. Pleural effusions are not commonly seen at breast US, unless they are large or saccular placed on the anterior chest wall. Furthermore, these findings occur mainly in the case of mastectomy, because the pleural line are more frequently included within the US FOV and these patients can have pleuro-pulmonary abnormalities more frequently. On US, pleura appears as echogenic band measuring up to 2 mm thick and, since this exam is dynamic, normal movement of the lung relative to the chest wall, “lung sliding sign”, should be recognized. Beyond the pleura-lung interface, the lung is air-filled and does not allow further visualization of normal lung parenchyma. The large change in acoustic impedance at this interface results in horizontal artifacts, seen as a series of echogenic parallel lines equidistant from one another below the pleural line; furthermore, also vertically oriented “comet-tail” artifacts can be normally seen, resulting from the fluid-rich subpleural interlobular septae surrounded by air [27].

**Fig. 12:**
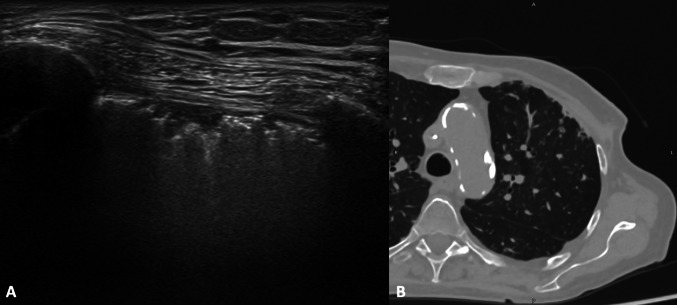
Female patient of 70 years old. B-mode US (**A**) and CT (**B**) scans. US demonstrates irregular pleural thickening as a hypoechoic band, superficial to the echogenic pleural-lung interface, suspicious of subpleural fibrosis in patient with mastectomy. CT confirms the diagnosis

Pleural thickening appears as hypoechoic broadening of the pleura, most frequently related to scarring, fibrosis, empyema, and pleuritis (Fig. 12). There may be an associated pleural effusion with or without increased vascularity at color Doppler [28].

**Fig. 13:**
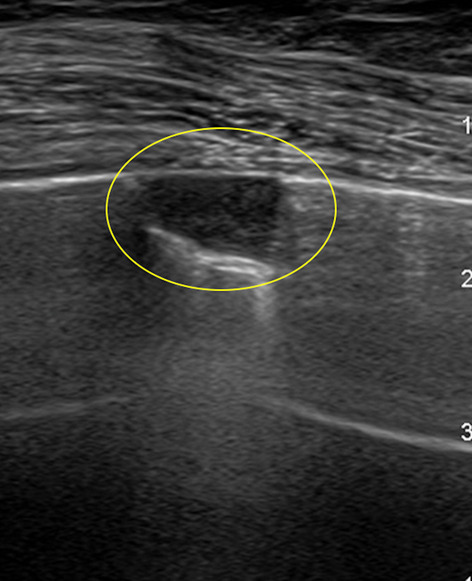
Female patient of 48 years old. B-mode scan. Pleural metastasis appears as a wedge-shaped hypoechoic nodule along the pleural line (yellow circle)

**Fig. 14:**
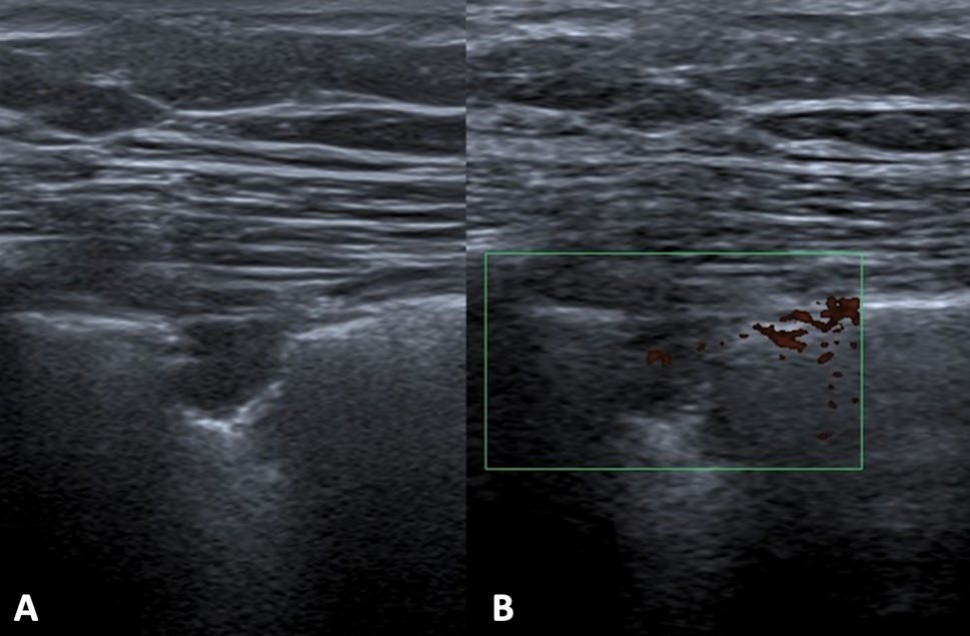
Female patient of 44 years old. B-mode (**A**) and power Doppler (**B**) scans. US shows a pleural metastasis as hypoechoic nodule, close to pleural line, characterized by irregular margins, posterior enhancement and intralesional vascularization on power Doppler

Pleural masses may be benign or malignant. The former, such as fibromas, lipomas, and neuromas, are uncommon, usually present as well-defined rounded masses of variable echogenicity, depending on the fat content. The latter include mesothelioma, lymphoma, and metastases. Mesothelioma is the most common primary malignancy of the pleura and asbestos exposure is the most important risk factor. The mean annual number of cases in Italy is 2.47/100,000 [29]. Mesothelioma appears at US as an irregular, nodular thickening of the pleura, frequently associated with a large pleural effusion. The pleural involvement with lymphoma may be primary or secondary. In details, the primary pleural lymphoma is extremely rare, especially in immunocompetent patients, whilst the secondary pleural lymphoma is very common, occurring in 20% of patients with lymphoma (non-Hodgkin lymphoma in 10 of cases) [30]. Subpleural lymphomatous deposits appear as wedge-shaped hypoechoic infiltrates. Finally, the most common pleural metastases are from primary adenocarcinoma. They can be identified as echogenic nodules more than 5 mm along the parietal or diaphragmatic pleura or as diffuse, irregular thickening of the parietal pleura (Figs. 13 and 14). Furthermore, malignant pleural disease may invade the chest wall, with poor demarcation of the pleural mass. Color-Doppler and pulsed-wave US may reveal neovascularity with irregular, tortuous vessels and low-resistance flow, respectively [31].

Pleural effusion appears as an echo-free layer between the visceral and parietal portion of the pleura [32].

Peripheral lung tumor appears as a homogeneous, well-defined mass, usually hypoechoic, but sometimes slightly echogenic, with posterior acoustic enhancement. US is more sensitive than CT for assessing invasion of the chest wall [33]; in particular, extension of the tumor beyond the parietal pleura into the chest wall can be confidently determined if the mass is seen to breach the pleura, with loss of sliding lung sign. Peripheral pulmonary metastasis present at US as multiple subpleural echogenic nodules measuring about 1–2 cm in diameter, high-vascularity on color-Doppler and low-resistance flow pattern on spectral Doppler [12].

**Fig. 15:**
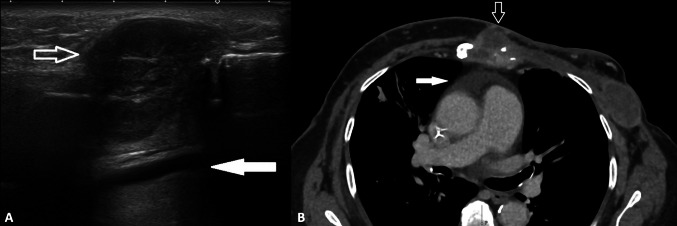
Female patient of 67 years old. B-mode US (**A**) and CT scans (**B**). Heterogenous and hypoechoic bone metastasis replaces the echogenic cortical line of the sternum in a patient with breast cancer (empty arrow in **A**). Deeper to the described lesion, anechoic pericardial effusion is observed (full arrow in **A**). CT confirms both pathological findings: bone metastasis (empty arrow in **B**) and pericardial effusion (full arrow in **B**)

Finally, pericardial effusion can sometimes be observed in the breast US FOV, it appears anechoic, deeper than the costochondral joint (Fig. 15) [34].

### Conclusions

It is important to remember that not all breast masses arise from breast itself: chest wall injuries may present as breast lump and thus be the reason for the examination. Furthermore, sometimes they may be an incidental finding during diagnostic or screening breast US. In both circumstances, radiologists must be able to adequately identify and characterize findings related to all structures included in the breast US FOV. The relatively risk-free noninvasive nature and fast examination time make US a useful screening tool, which can aid in determining whether a lesion is present, where it is located, and whether it is cystic or solid. Color-Doppler and spectral tracings can provide additional information regarding vascular flow. On the other hand, the inability to see deeper structure and to penetrate bone limits the usefulness in assessing carefully lesions that originate from chest wall structure. In these cases, CT and MRI are complementary imaging techniques that provide information about disease nature and extent.
